# Meet Our Editorial Board—*Engineering in Life Sciences*. An Interview with Antonina “Tonya” Lavrentieva, Leibniz Universität Hannover, Institut für Technische Chemie, Hannover, Germany

**DOI:** 10.1002/elsc.70014

**Published:** 2025-03-17

**Authors:** Paul Trevorrow, Antonina Lavrentieva

**Affiliations:** ^1^ Wiley, The Atrium, Southern Gate Chichester UK; ^2^ Leibniz Universität Hannover Institut für Technische Chemie Hannover Germany


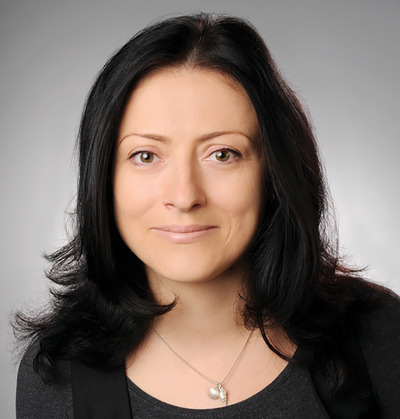


Antonina Lavrentieva is a group leader of Cell Culture Technology at the Institute of Technical Chemistry, Leibniz University of Hannover, working in the field of stem cell research, 3D cell culture and bioprocess development for cultivated fat production. In 2022 she received the *venia legendi* in Technical Chemistry. In her second PhD Thesis, she studied methods of expanding mesenchymal stem cells (MSCs) in bioreactors, as well as the influence of hypoxia on the MSCs. She studied Biology and Life Science at Moscow State University and the Leibniz University of Hannover. She also defended a PhD Thesis in Physiology. Her current research interests include stem cell media optimization, 3D cell culture, implementation of genetically encoded sensors for 3D cell culture characterization, gradient hydrogels for studying stem cell niches and cellular agriculture, particularly cultivated culinary fat. Currently, she is the head of advisory board of Deutsche Gesellschaft für Chemische Technik und Biotechnologie (DECHEMA) professional group “Medical Biotechnology”.

**Would you briefly explain what your research group is studying?**

As a group leader in cell culture technology, my team focuses on three main topics. First, we develop 3D cell culture systems by synthesizing various hydrogels and analyzing cell growth within them. Second, we modify cells with genetically encoded biosensors to monitor behaviors such as hypoxia and apoptosis. Third, we have recently begun developing bioprocesses for cultivated fat, working with the Berlin/Hannover‐based startup Cultimate Foods to isolate and expand porcine and bovine stem cells in bioreactors.

**How did you choose a career in biotechnology?**

I have two PhDs. The first one was in biology, which I studied at Moscow State University, followed by a PhD in neuroscience. Although the first PhD was successful, I decided not to continue working with experiments, in part, because it involved the use of many laboratory rats. When I relocated to Germany, I sought a more application‐focused field. Consequently, I earned a Master of Science in life science and subsequently completed a second PhD in biochemistry, specifically in technical chemistry, which is also known as chemical engineering. In this field, we primarily focus on various types of biotechnology. Ultimately, I also obtained habilitation in chemical engineering. Thus, my background is rooted in biology, but I have transitioned to biotechnology, working closely with chemists and engaging in cell culture research.

**What excites you the most about the field and why?**

Biotechnology is incredibly versatile, offering something for everyone. I am particularly fascinated by the wide array of bilogical tools and processes we can harness use to solve complex problems. We can learn so much from nature, with many discoveries still ahead. Although I am passionate about my specific area, the field of biotechnology as a whole is widely interesting.

**What are your favorite pastimes outside of research? What do you like to do for fun?**

I work in my garden, grow heirloom tomatoes. I collect and exchange seeds, which is technically illegal in Germany due to an old law from the 1930s that restricts growing certain types of tomatoes. It was a forgotten and ridiculous regulation. Besides gardening, my husband and I enjoy hiking and kayaking. We prefer outdoor activities for exercise.

**If you had 1 year where you had no work, and you had as much money as you ever wanted, what would you do?**

If I had the time and money, I would travel. I would visit places that are usually too far or require long flights. For example, I dream of taking a long cruise to Antarctica when I retire. I would love to travel and stay at each destination for more than just a couple of days.

